# The ulna osteotomy locking plate II in patients with ulnocarpal impaction syndrome: a retrospective evaluation

**DOI:** 10.1007/s00402-026-06223-5

**Published:** 2026-03-17

**Authors:** Stefan Benedikt, Ulrike Seeher, Moritz Stricker, Simone Bode, Kerstin Stock, Rohit Arora

**Affiliations:** Department of Orthopaedics and Traumatology, Innsbruck Medical University, Innsbruck, Austria

**Keywords:** Shortening, Tolat, Malunion, Ulnocarpal impaction syndrome, Ulnar wrist pain, TFCC, Discus

## Abstract

**Introduction:**

Ulnocarpal impaction syndrome is one general cause of ulnar-sided wrist pain. If conservative therapy fails, various surgical procedures and different instruments and implants are available. The aim of this study was to evaluate the results of the ulna osteotomy locking (UOL) plate II from the manufacturer I.T.S.

**Materials and methods:**

Thirty-five cases (34 patients) with primary or secondary ulnar impaction syndrome treated with the UOL plate II were evaluated retrospectively. Demographic, radiologic and clinical data were collected focusing on the postoperative ulnar variance and the complication rate, especially non-unions and signs of implant failure.

**Results:**

Median age was 48 years, with 20 female and 15 male cases. The measured median postoperative ulnar variance was − 0.8 mm (Q1: − 2; Q3: 0). There was no case of a non-union or a postoperative infection. Postoperative complications included one case of secondary dislocation, one case of a neuroma of the dorsal branch of the ulnar nerve and one mild CRPS. A total of six cases wished for an implant removal in the follow-up, of which five complained about local irritation caused by the plate.

**Conclusions:**

The UOL plate II has proven to be a reliable option for ulnar shortening osteotomy with a low implant associated complication rate, especially no case of non-union. The occasional need for implant removal should be considered. Due to the retrospective study design, a prospective study comparing the UOL II plate to different plating systems would be desirable.

## Introduction

Ulnocarpal impaction syndrome is one general cause of ulnar-sided wrist pain. It may either occur idiopathic in patients with positive ulnar variance, as a dynamic impaction even in patients with a neutral or negative ulnar variance [39], or posttraumatic e.g. after distal radius fracture malunions in up to 18% [33].

Diagnosis should be based on the assessment of medical history with special regards to past injuries, followed by a thorough clinical examination and verified with appropriate imaging [43].

Key clinical tests are the ulnocarpal stress test [25] and a positive ulna fovea sign [37], while examination for ECU-tendon pathologies should be performed to clinically rule out relevant differential diagnoses [30]. Special consideration should be payed to distinguishing symptoms caused by DRUJ instability, which demands a different treatment [13].

Basic imaging consists of static a.p. and lateral wrist x-rays followed by pronated grip views, especially if there is no apparent positive ulnar variance in the standard view, to become aware of dynamic ulnar impaction [11, 32, 40]. MRI imaging should be obtained in all patients, with findings such as increased signal intensity in the lunate, triquetrum or ulnar head and thinning or perforation of the central portion of the TFCC confirming the diagnosis [43, 45]. In addition, concomitant pathologies such as cartilage damage can be displayed. In patients with contraindication for MRI imaging, SPECT/CT imaging is a feasible alternative [36]. In case of concomitant injuries, especially suspected TFCC injury, wrist arthroscopy is a well established diagnostic tool [41].

The recommended first line treatment is conservative, including splint immobilisation, pain medication and rehabilitation, and is successful in up to 59% of patients [29]. If this does not lead to long term success, there exists a variety of surgical treatment options which should be considered.

The surgeon may choose from arthroscopic procedures such as debridement and synovectomy, wafer procedures to ulnar shortening osteotomies, for which in turn there is a range of products and techniques on offer [21, 24, 35].

One available solution is the Ulna osteotomy locking plate II (UOL II, Fa. I.T.S., Autal 28, 8301 Lassnitzhöhe/Graz, Österreich). It is a single device system to perform the placement of the anatomical plate, osteotomy and compression. A transection gauge is temporarily fixated on the plate and guides an oblique osteotomy in the distal third of the ulnar shaft, allowing an ulna shortening up to 8 mm in one step or up to 16 mm in two steps. The plate can be equipped with locking or compression screws as well as a lag screw perpendicular to the osteotomy.

In this study, we examined the outcomes after ulna shortening osteotomies with the use of the UOL II plate.

## Patients and methods

Approval for conducting this study was obtained by the institutional review board (no. 1061/2025). Inclusion criteria were patients with an ulnocarpal impaction syndrome treated between January 2016 and October 2024 with the UOL II Plate at the Department of Orthopaedics and Traumatology at the University Hospital of Innsbruck.

Patients that required any osteotomy of the distal radius at the same time were excluded. Study design was a monocentric retrospective data evaluation. All available documents and medical imaging of the hospital information system were used for this purpose. Retrospective analysis and measurements were performed by two experienced residents.

Analysed demographic data included age, sex, smoking behaviour and aetiology. Further parameters were follow-up time, pre-existing medical conditions or comorbidities as well as long-term medication that may have a negative impact on bone healing (Table 1). Preoperative X-rays were assessed for ulnar variance (method of perpendiculars [34]), palmar tilt (Böhler II [42]), radial inclination (Böhler I [42]) and configuration of the DRUJ (Tolat type [38]).

**Table 1:** Analysed preoperative conditions/comorbidities and medication that have a negative impact on bone healing [14, 27]

Comorbidities	Medication
Cancer	Chemotherapy Hormone therapy
Osteoporosis	Bone-loss therapy
Hypothyroidism	Thyroid hormone
Rheumatoid disease	Steroids Non-steroidal anti-rheumatic drugs
Peripheral vascular disease	Low-molecular heparin
Malabsorption (e.g. Chron. Colitis)	
Diabetes	
	Anticonvulsants
	Antibiotics

Data regarding surgery included operation time, ulnar shortening length according to the surgical report, additional procedures (e.g. arthroscopy), type of immobilisation, and usage of a lag screw (yes/no).

Outcome data included the non-union rate, immobilisation time, postoperative ulnar variance, complications such as implant loosening and fragment dislocation, duration of hospitalisation, subjective outcome, and rate of implant removals.

The descriptive statistic was performed with SPSS (version 29, IBM SPSS Statstics, 1 Orchard Road, Armonk, New York, United States). Due to the suspected low sample size between 30 and 40 cases the median and the first and third quartile (Q_1_ and Q_3_) were used for data evaluation.

### Surgical technique

**Fig. 1:**
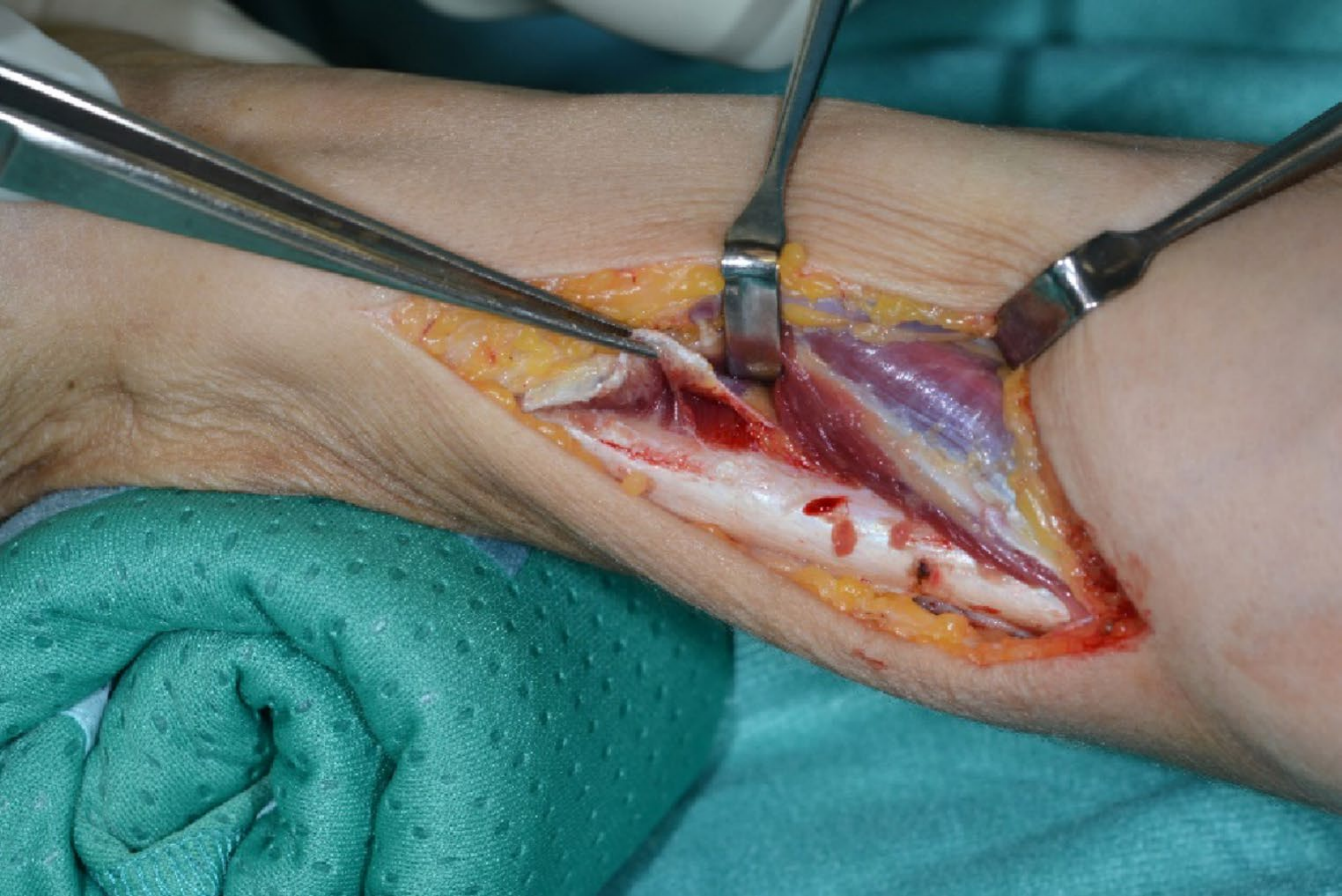
Approach to the distal ulna with the pronator quadratus muscle detached at the ulnar insertion (forceps)

Surgery was performed in general or plexus anaesthesia in supine position. All patients received a single-shot antibiosis. Skin incision was performed 2 to 3 cm proximal to the tip of the ulnar styloid running 8 cm proximally along the ulnar margin of the forearm. The forearm fascia was incised and the bone was approached between the extensors dorsal and the flexors palmar. The pronator quadratus muscle was detached. The periosteum was left on the bone to preserve blood supply (Fig. 1).

**Fig. 2:**
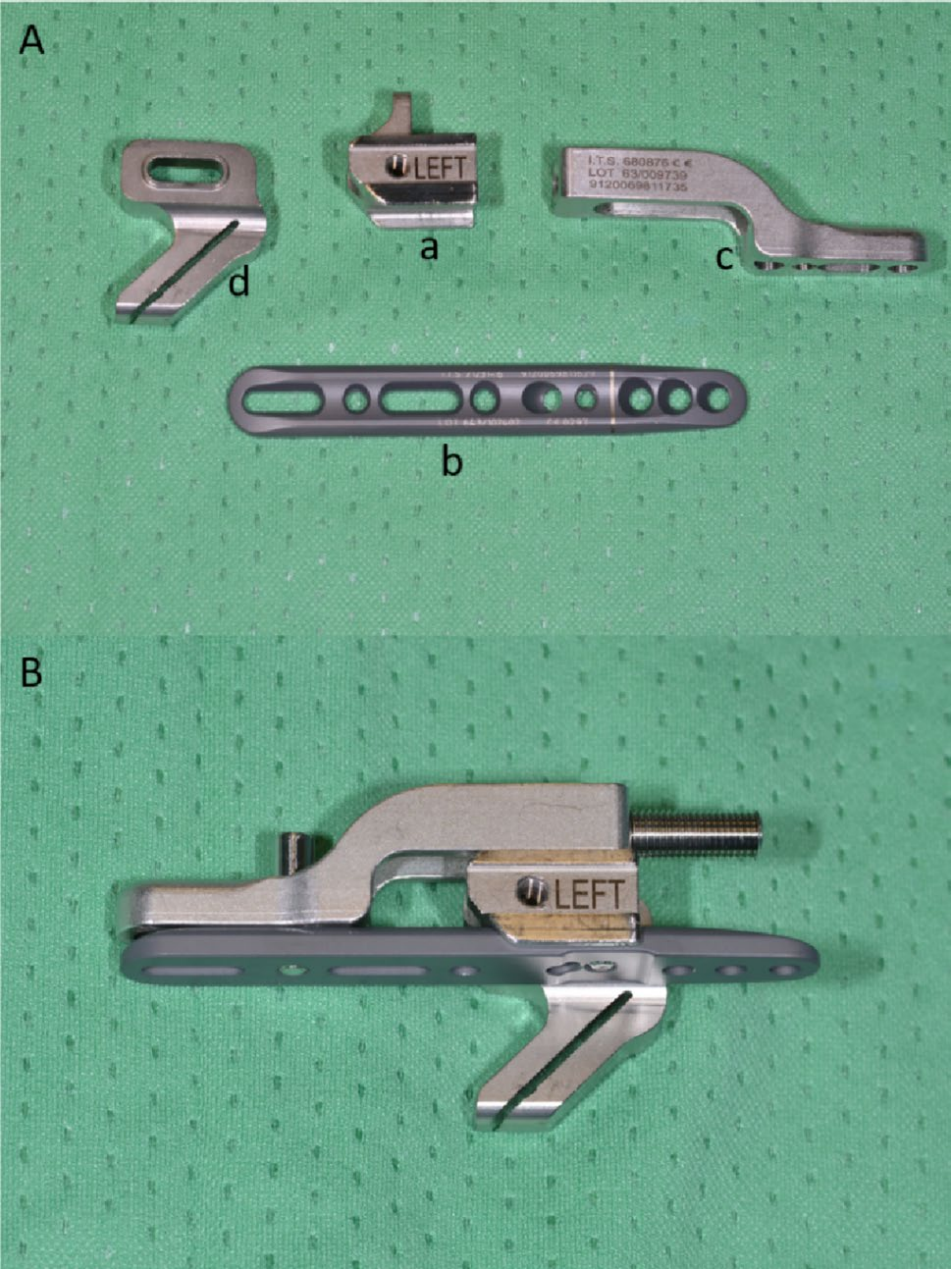
**A** First, the holder (a) is fixated on the plate (b) with a fixing screw. The compression slide (b) is then fixated on the plate (b) using a fixing screw. Finally, the cutting gauge (d) is fixated with a screw to the holder (a). **B** The ready-assembled instrument (the compression screw at the top right has already been inserted for demonstration purposes but should not be assembled before the plate is fixed distally)

The components and assembly of the instrumentation are shown in Fig. 2.

**Fig. 3:**
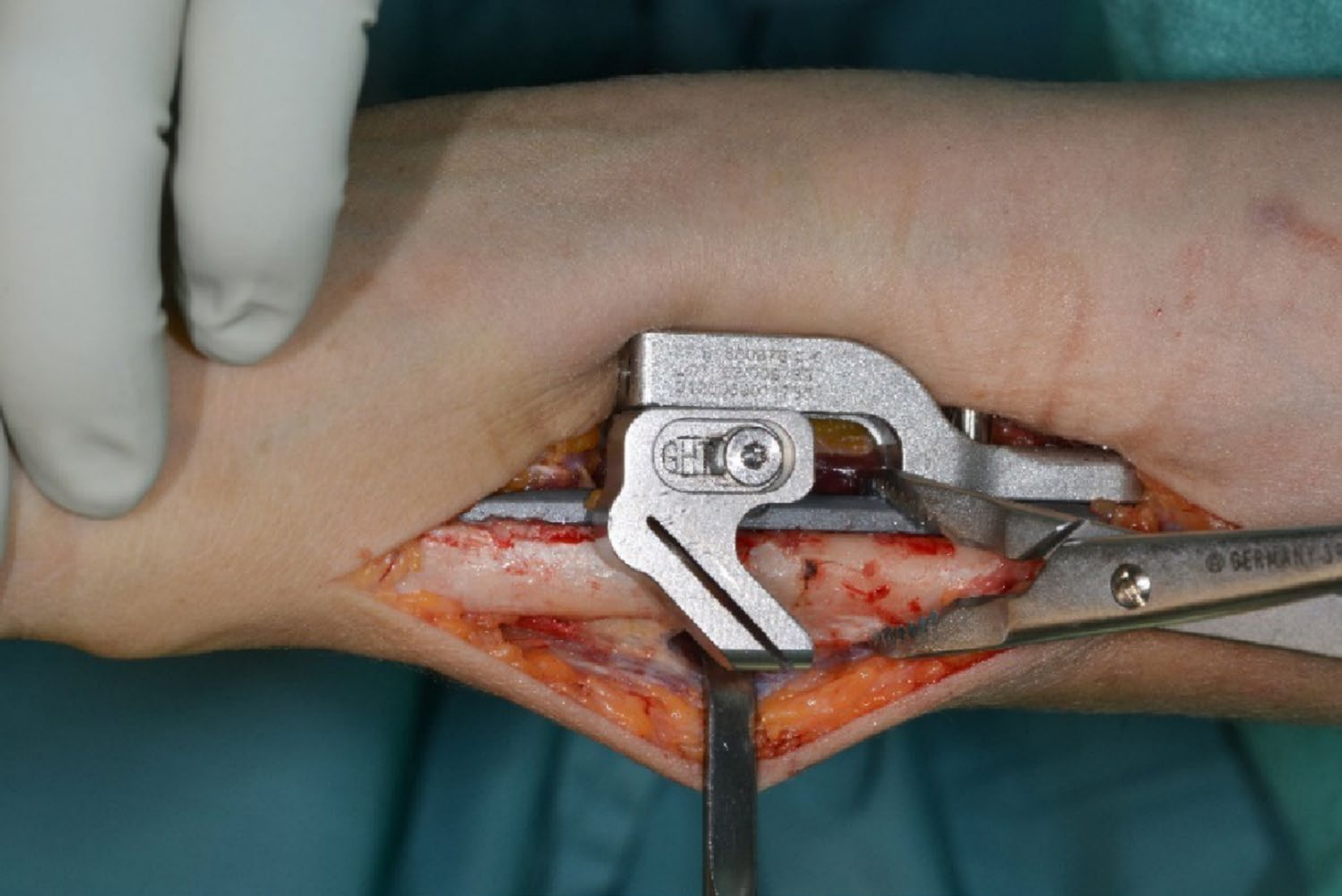
Positioning of the osteotomy device palmar on the distal ulna

The device was fixated palmar to the bone via a clamp and position was checked clinically and via image intensifier. The distal plate holes were inserted with bicortical angular stable screws (Fig. 3). Two tension bolts were inserted proximally to connect the compression slide to the bone in a stable manner but still allow sliding between the compression slide and the plate for later compression of the osteotomy.

**Fig. 4:**
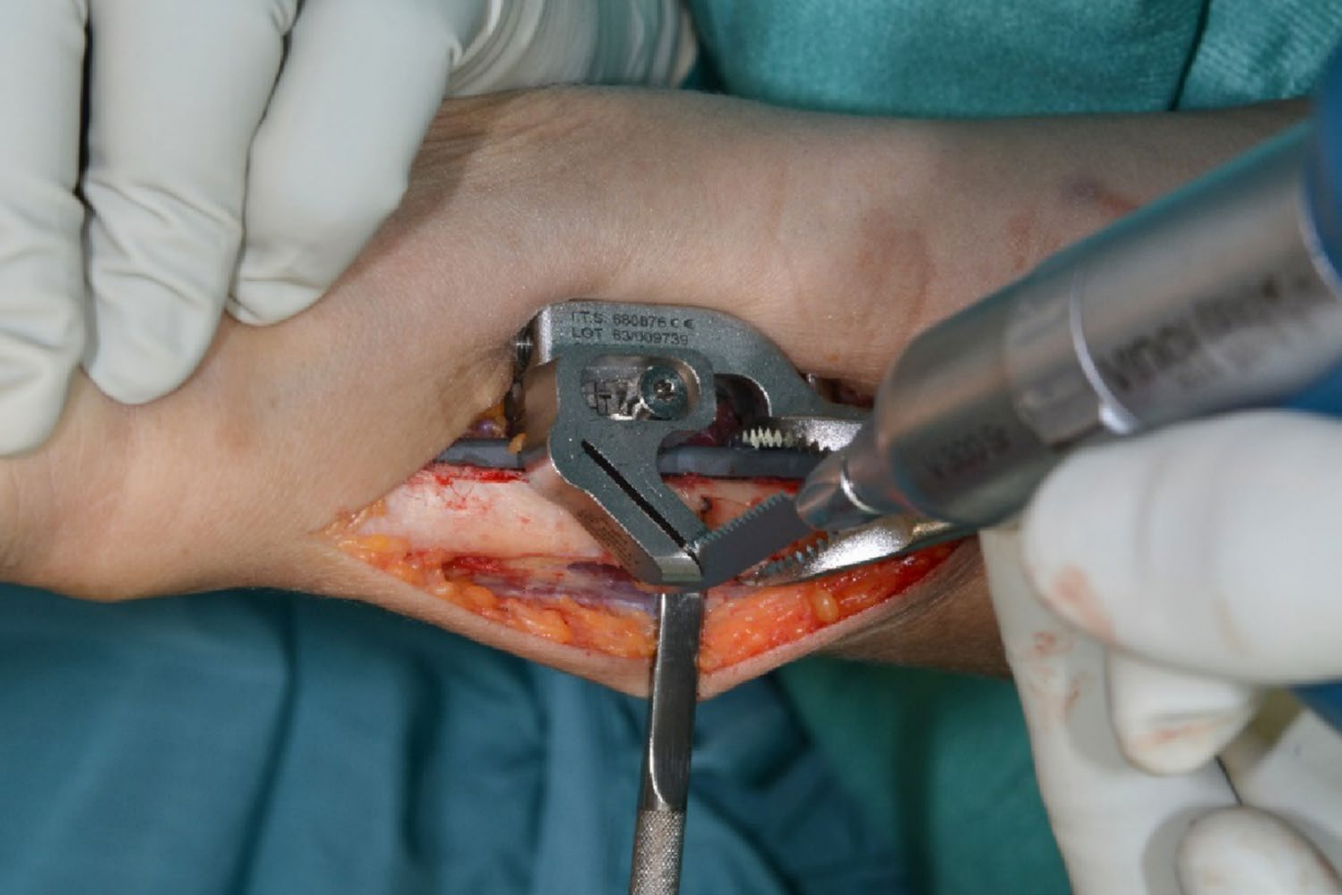
Oblique osteotomy after retraction of the periosteum

**Fig. 5:**
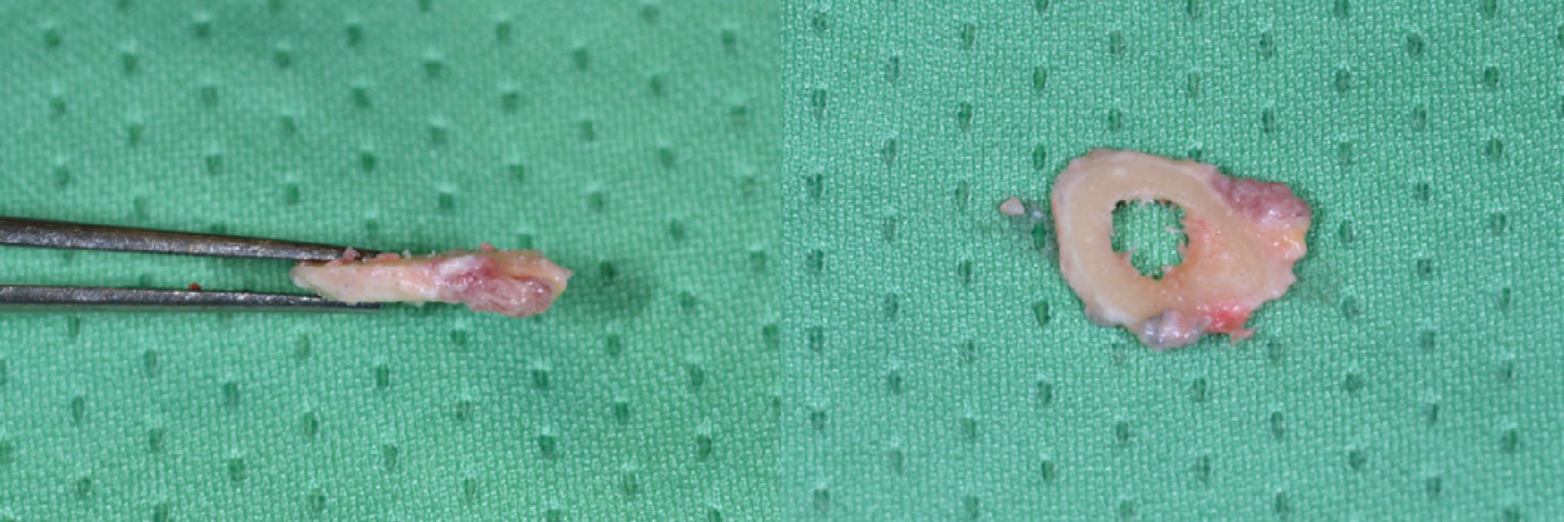
Resected bone slice

**Fig. 6:**
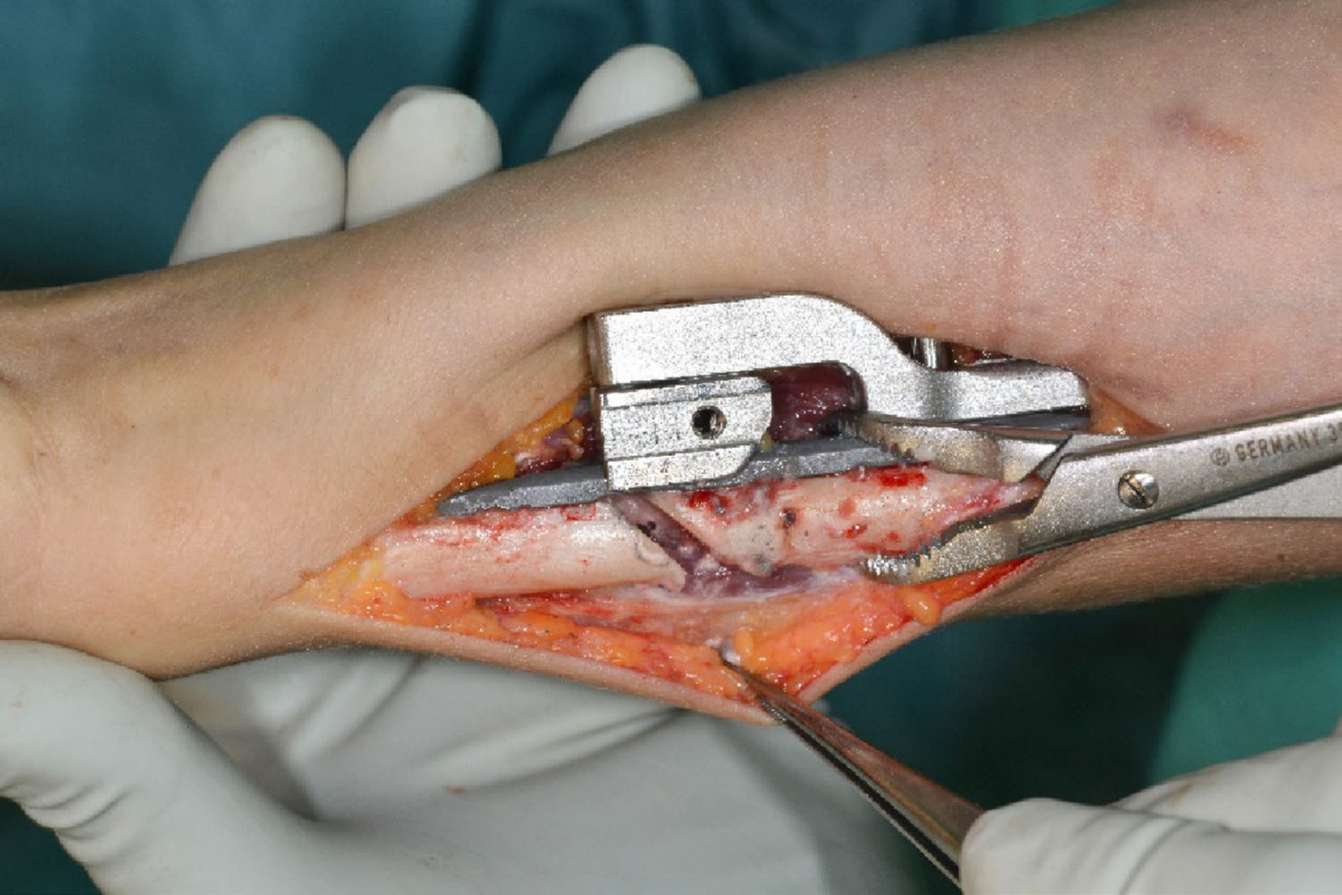
Distal ulna after osteotomy

The periosteum was incised at the level of the planned osteotomy and minimally retracted. Then the first oblique osteotomy at 0 mm and the second at the desired osteotomy length were performed (Figs. 4, 5 and 6).

**Fig. 7:**
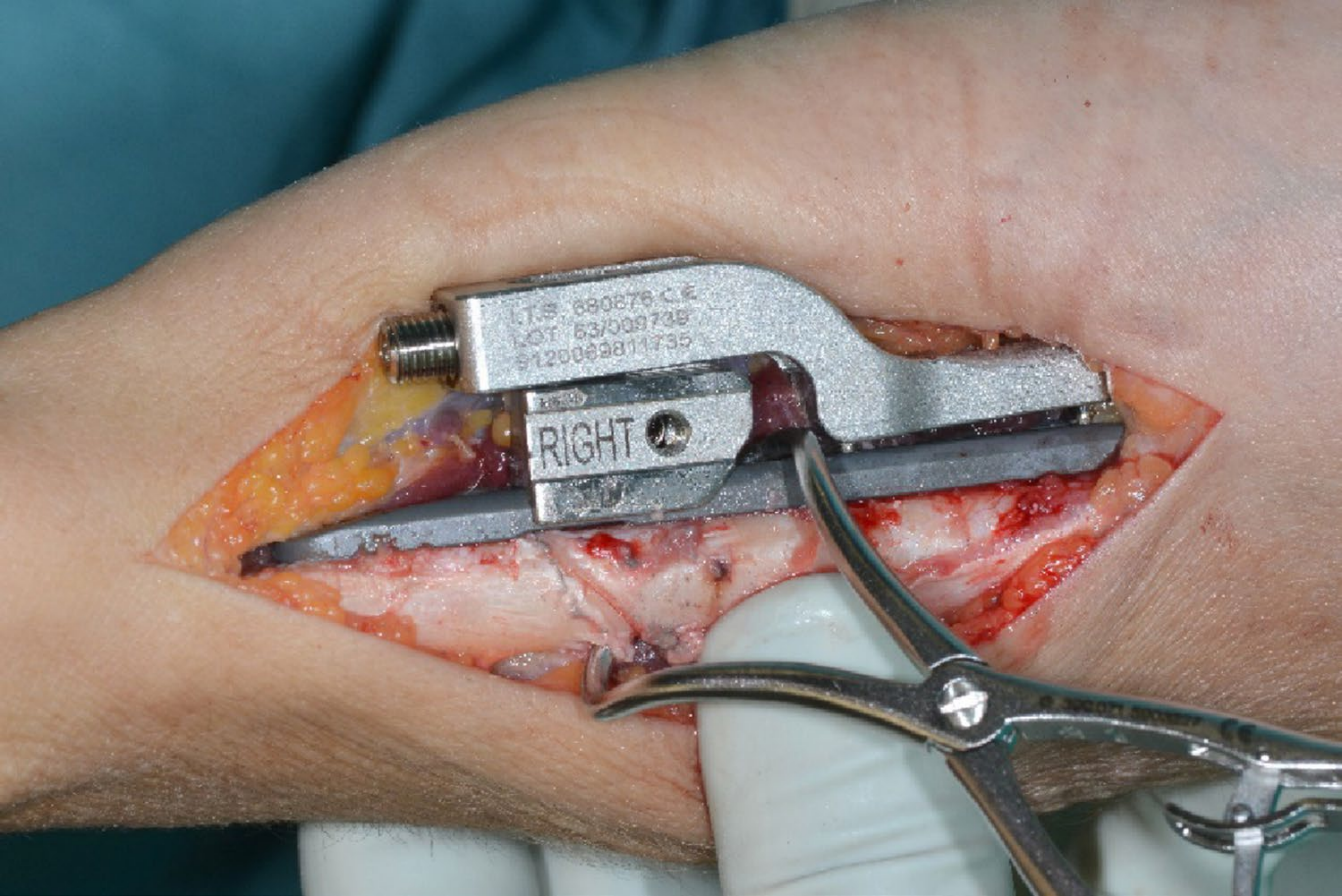
The osteotomy gap is approximated via the compression screw and secured with a reduction clamp

**Fig. 8:**
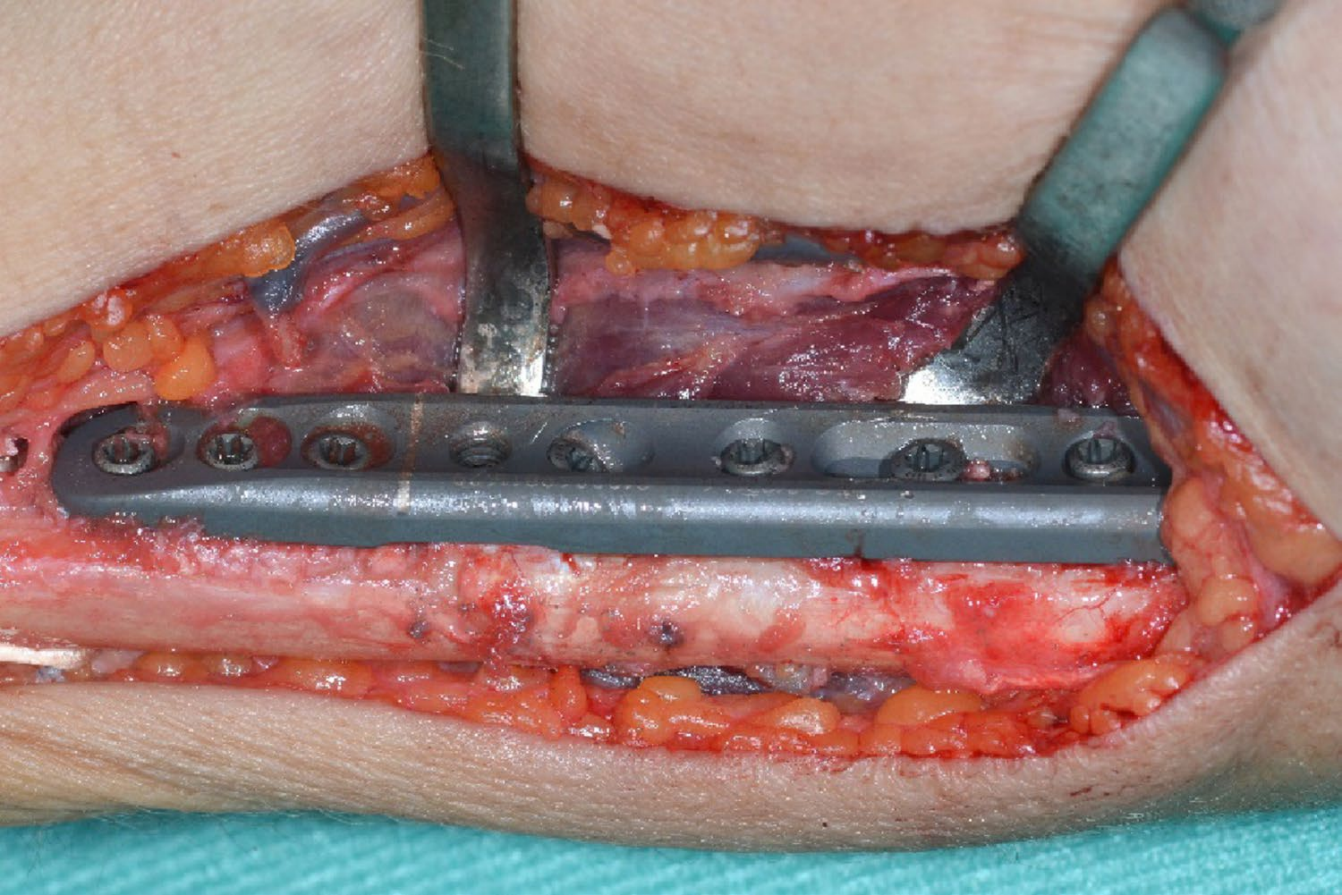
Plate with all screws inserted

The dissection gauge was removed and the osteotomy gap was then closed using the compression screw and secured with a reduction clamp. Interfragmentary compression was applied with a lag screw via the corresponding 45 degree drill hole of the device (Fig. 7). The proximal screw holes were inserted with bicortical screws and the osteotomy device was removed (Fig. 8).

## Results

### Demographic data

A total of 35 forearms of 34 individuals was included (one bilateral osteotomy), of which 20 cases were female and 15 male. Median age was 48 years (Q_1_: 30; Q_3_: 55). Seven of 35 cases stated to be smoker, 25 were non-smoker. Comorbidities included one case each of the following: colitis, diabetes mellitus type 2, lupus erythematosus, and osteoporosis, while three cases presented with hypothyroidism, and one case with a combination of hypothyreodism, osteopenia and celiac disease. Long-term medication included osteo-anabolic treatment (teriparatid) and an estrogen/gestagen substitution in respectively one case, while all cases with hypothyroidism received a substitution therapy. Twenty cases (57.1%) stated a previous trauma in the past medical history of which 12 cases (34.3%) sustained a fracture (10 distal radius fractures, one essex-lopresti injury and one Galeazzi fracture). Two cases had a Madelung deformity. The preoperative median ulnar variance was + 2 mm (Q_1_: 1; Q_3_: 3), the median radial inclination 23° (Q_1_: 20; Q_3_: 25), and the median palmar tilt 8.9° (Q_1_: 6; Q_3_: 16). 29 cases had a tolat type 1 configuration while we found a tolat type 2 and 3 configuration each in 3 wrists. Median follow-up was 8 months (Q_1_: 4; Q_3_: 18).

### Surgical/operation data

Median operation time was 90 min (Q_1_: 72; Q_3_: 115). The median shortening was 3 mm (Q_1_: 2; Q_3_: 4) according to the surgical report. The lag screw was used in all cases. We performed additional arthroscopy in 18 cases including 4 TFCC stabilisations. Other additional procedures included one resection of a carpal boss and arthroscopic removal of chondrocalcinosis, one removal of an ulnar loose body, one arthroscopic resection of a synovial cyst, one local cancellous bone graft in an incongruent osteotomy gap, two implant removals of the distal radius, one sympathectomy at the upper arm in a case with chronic pain syndrome, one release of the first extensor tendon compartment and one neurolysis and transposition of the ulnar nerve in combination with an arthrolysis and implant removal at the elbow. 30 cases were postoperatively immobilised with a forearm cast, while one patient did not receive any immobilisation. In the four cases with the TFCC stabilisation, immobilisation with a sugar-tongue splint was performed.

### Postoperative outcome

**Fig. 9:**
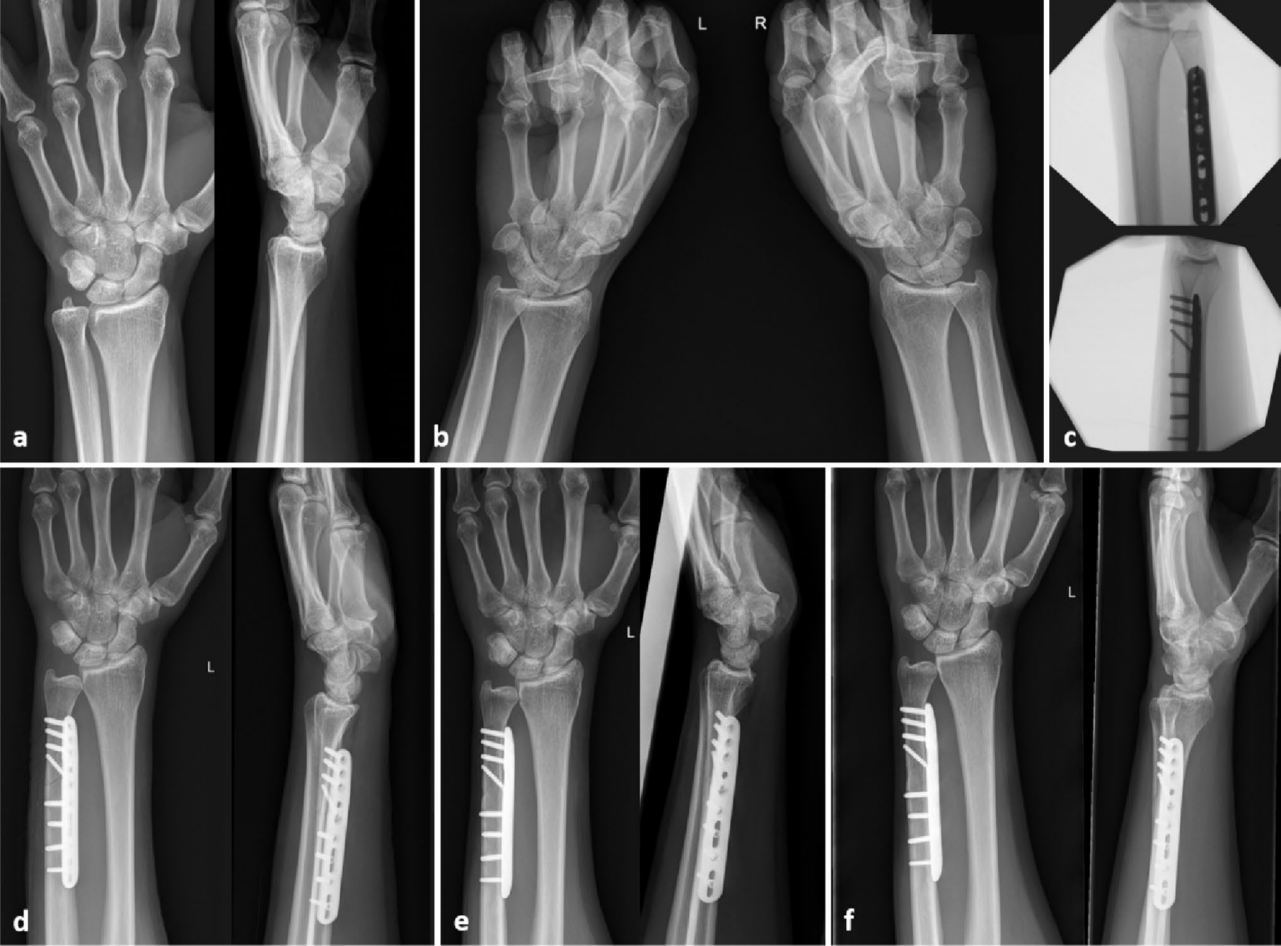
Case with a dynamic ulnar impaction syndrome successfully treated with the UOL Plate II. **a** X-ray preoperatively, **b** clenched fist X-ray preoperatively showing a dynamic positive ulnar variance on both sides, **c** intraoperative fluoroscopic control, **d–f** postoperative X-ray two weeks, three and six months postoperativelyshows an example of a case with a left sided dynamic ulnar impaction syndrome treated with the I.T.S. UOL System II

Figure 9 shows an example of a case with a left sided dynamic ulnar impaction syndrome treated with the I.T.S. UOL System II.

Overall, there was no case of a non-union or a postoperative infection. The median forearm cast immobilisation was 26 days (Q_1_: 23; Q_3_: 29). The sugar-tongue cast immobilization ranged from 40 to 44 days. The measured median postoperative ulnar variance was − 0.8 mm (Q_1_: -2; Q_3_: 0). The median duration of hospitalisation was 3 days (Q_1_: 2; Q_3_: 3). Twenty-four cases received non-steroidal anti-rheumatic drugs after the surgery.

Three surgery-associated complications were documented: One case had a secondary dislocation of the distal ulnar due to technical problems and was revised with reosteosynthesis. In one case, a neuroma of the dorsal branch of the ulnar nerve was observed about one year after osteotomy and revised with neurolysis, nerve transposition and lipofilling. One case developed a mild CRPS.

As many patients were lost during mid-term follow-up, anamnestic subjective outcome could only be assessed to a limited extend. Subjective outcome data of 11 cases were missing. 18 cases stated an improvement and were satisfied regarding the ulnar shortening while six cases had persisting complaints: In two cases the complaints were more likely due to a symptomatic pisotriquetral synovial cyst and a chronic pain syndrome, one case was the patient with the reported neuroma of the dorsal branch of the ulnar nerve. The other three cases had a previous trauma including two distal radius fractures. One of them was treated with a TFCC stabilisation, one with arthroscopic debridement at the time of ulnar shortening. The sigmoid notch of all patients with persistent complaints was classified as Tolat type 1. The palmar tilt of the distal radius of these six cases ranged between − 16.3 and 20.0°, the shortening length ranged from 2 to 4 mm. The case with the palmar tilt of 20.0° had a primary UIS without trauma.

A total of six cases wished an implant removal in the follow-up of which five complained about local irritation caused by the plate.

## Discussion

The UOL plate II system has proven to be a reliable option for ulnar shortening osteotomy with a low complication rate. Especially, no cases of non-union were found, although patients with several risk factors for impaired bone healing were documented. Apart from the case of secondary dislocation, the other two cases can be considered general complications and could in theory also occur with other implants or operations. While 18 cases stated improvement, six had persistent complaint. However, all these six patients had either other comorbidities or a trauma in the past medical history which could have influenced the clinical results.

The USO is a long-established and highly effective method for treating ulnar impaction syndrome. Nevertheless, primary treatment of ulnocarpal impaction syndrome should be conservative; if this does not prove to be effective, a surgical approach should be considered [43]. 

Surgical alternatives to USO are the open and arthroscopic wafer procedure that should only be considered in an ulnar-positive variance of up to 2–3 mm [43]. Even though numerous studies have addressed this topic, there is no clear evidence for the superiority of one surgical technique or the other. Although patient satisfaction rate is slightly higher in either open (89%) or arthroscopic (100%) wafer procedure compared to USO (84%), the number of published cases of ulnar shortening osteotomy is significantly higher and still seems to be the surgeons’ most common choice [35]. While the wafer procedure is limited to a reduction of up to 2–3 mm, its advantages clearly lie in being minimally invasive with a similar effective pain relief and restauration of function, while there is no risk for non-union and no need for postoperative cast immobilisation or hardware removal [8]. Although the implant design allows for an ulnar shortening of up to 16 mm, median shortening in our study was 3 mm, leading to a median postoperative ulnar variance of -0,8 mm, compared to + 2 mm preoperatively. This aligns with other reported average shortenings ranging from 2 mm up to 5,3 mm [2, 5, 6, 10, 15, 19, 26]. This implies that the limitation of shortening in the wafer procedure may not be as relevant as previously discussed [7].

Arthroscopic debridement of the discus as a surgical first line therapy in Palmer 2 C lesions is also described to be a successful alternative, especially in patients with an ulnar variance up to 1.8 mm, thereby being even less invasive than the wafer procedure [24].

Radial lengthening osteotomy after distal radius fracture malunion should be considered especially in young patients with a dorsal tilt > 10° or palmar tilt > 15° and good bone quality. It may lead to better pain reduction and improvement of function but is also the more complex and time-consuming procedure [1, 23].

In the earlier years of the USO there was uncertainty as to the best type of osteotomy (transverse, oblique, or step cut), as non-union rates deemed higher in transverse cuts and were reported up to 16% [20, 28]. However, a multitude of more recent studies has proved non-union rates to be consistently low (0–6%) regardless of the osteotomy type [5, 6, 10, 16, 18, 19, 26]. These findings are in accordance with the result of our study, where a 0% non-union rate with an oblique osteotomy and interfragmentary compression was found.

While some authors credit low non-union rate to more specialised implant design [6, 22], others have shown non-union rates to be equally low with standard 3,5 mm plates or 1/3 tubular plates [2, 26] while at the same time being more cost-efficient.

Cha et al. identified smoking, low bone mineral density, a decreased ROM of the wrist, and use of a double-blade saw as prognostic factors for non-union [4]. While the latter remains irrelevant for this study due to osteotomy design, we assessed the patients for smoking habit (*n* = 7) and prevalence of osteoporosis (*n* = 1) but did not encounter any case of non-union. However, this may well be linked to a substantially larger study group of 325 patients in Cha et al., which provides a better foundation for analysis.

The UOL II plate is a diaphyseal implant. A meta-analysis by Deng et al. in 2023 [9, 17] evaluated the effectiveness of metaphyseal and diaphyseal USO in UIS. Metaphyseal USO showed significantly lower second operation rates while also providing slightly better pain relief and DASH scores. However, the incidence of complications and the difference of grip strength is not significant between both techniques. Deng. et al. [9, 17] recommend the metaphyseal shortening osteotomy especially for patients with osteoporosis, non-union risk and smoking history, however in our study population, none of these factors had a negative impact on infection or non-union rate. In metaphyseal USO, the opening of the distal radioulnar joint and the possible resulting joint stiffness or injury to the discus may be considered a disadvantage [17]. On the other hand, a clear disadvantage of the diaphyseal USO is the occasional need for implant removal and the associated fracture risk after removal while in metaphyseal USO the screw can be buried in the bone [17]. In our study, implant removal was performed in six patients (17%), which is similar to lower compared to most studies that report an implant removal rate of 7–67% [5, 10, 16, 22, 26]. However, Clark et al. reported an implant removal rate of 0% in a cohort of 93 wrists after an average follow-up of 6 months with the usage of the Acumed low-contact plate (Acumed, Hillsboro, USA), crediting this to the good soft tissue coverage of the low profile when placed volar on the ulna [6]. An additional explanation might be that implant removal is in general more common in Austria than the United States, supposedly due to public financing [44]. There were no fractures after implant removal among our patients.

Perseverant discussion revolves around the DRUJ, suggesting that Tolat type 3 may be unsuited for USO. Sagerman et al. showed a significant correlation between the inclination of the sigmoid notch and the ulnar variance and directed the attention to the impact USO may have on the DRUJ [31]. Although in the original work by Tolat et al. Type I was the most common DRUJ configuration (55%) followed by Type II (33%) and Type III (12%) [38], Baek et al. performed USO in a vast majority of Type III DRUJ (*n* = 26, 72,2%) [2]. The high prevalence of Type III DRUJ may be linked to the correlation with a positive ulnar variance, leading to UIS requiring the need for surgery. Baek et al. postoperatively found radiographic signs of osteoarthritis in 19% of the Tolat Type III wrists, without any clinical complaints. In a cohort of 62 patients, Gilbert et al. were able to show better results regarding supination, grip-strength and DASH-Scores in Tolat Type I wrists, while not witnessing any onset of osteoarthritis after a minimum follow-up of 5 years in any Tolat type [12]. This is supported by the findings of Cha et al., who did not find the morphological DRUJ type a significant factor for arthritic changes in a total of 310 patients after USO [3]. In the authors’ opinion, this does not provide enough evidence to define Tolat Type III as a contraindication for USO. In our study, this is supported by the finding that none of our cases with persistent complaints had a Tolat Type III DRUG. A downside to our study is the lack of grip strength and DASH-Score-measurements to provide further evidence in this matter.

Few authors report on operation time. Compared to the published operation time of an average of 39 min by Elgammal et al. (*n* = 27) and 58 min by Clark et al., we took considerably longer with a median operation time of 90 min [6, 10]. We believe this is due to the high amount of concomitant procedures, especially arthroscopic procedures in 18 of the 35 wrists, including time consuming surgeries such as arthroscopic TFCC stabilisations. None of the complications we encountered could be linked to a long operation time.

Limitations of this study include the low number of cases, exaggerated by lost follow-ups due to the retrospective study design. Moreover, ulnar shortening osteotomy was combined with other concomitant procedures in numerous cases, making the study population inhomogeneous. Clinical outcome measures as range of motion and grip strength as well as subjective functional outcome scores were not assessed. A prospective study comparing the I.T.S. ULP II plate to different plating systems in regard of clinical and radiological outcomes as well as time and cost efficiency would be desirable.

## Conclusion

Among the numerous treatment options for ulnar impaction syndrome, each procedure has its indications, advantages and disadvantages. The treatment plan should always be adjusted to the patient’s expectations and requirements.

Ulnar shortening osteotomy remains a recommended standard treatment for ulnocarpal impaction syndrome [43]. When it comes to the choice of a specific implant, the UOL II has proven to be a valuable therapy option. Except for the one case of secondary dislocation no other implant associated complication or non-union occurred. The occasional need for implant removal should be considered.

## Data Availability

The data that support the findings of this study are available on request from the corresponding author.
